# The first genetically confirmed cohort of Facioscapulohumeral Muscular Dystrophy from Northern India

**DOI:** 10.1038/s41431-024-01577-z

**Published:** 2024-04-25

**Authors:** Venugopalan Y. Vishnu, Richard J. L. F. Lemmers, Alisha Reyaz, Rinkle Mishra, Tanveer Ahmad, Patrick J. van der Vliet, Marcelina M. Kretkiewicz, William L. Macken, Stephanie Efthymiou, Natalia Dominik, Jasper M. Morrow, Rohit Bhatia, Lindsay A. Wilson, Henry Houlden, Michael G. Hanna, Enrico Bugiardini, Silvère M. van der Maarel, M. V. Padma Srivastava

**Affiliations:** 1 Department of Neurology, All India Institute of Medical Sciences (AIIMS), Delhi, India; 2 Department of Human Genetics, Leiden University Medical Center (LUMC), Leiden, The Netherlands; 3 Department of Neuromuscular Diseases, Queen Square Institute of Neurology, University College London, London, UK; 4 NHS Highly Specialised Service for Rare Mitochondrial Disorders, Queen Square Centre for Neuromuscular Diseases, The National Hospital for Neurology and Neurosurgery, London, UK; 5 Department of Neuromuscular Diseases, Queen Square UCL Institute of Neurology and the National Hospital of Neurology and Neurosurgery, London, UK

**Keywords:** Genetics research, Neuromuscular disease

## Abstract

Facioscapulohumeral muscular dystrophy (FSHD) is the third most common form of hereditary myopathy. Sixty per cent of the world’s population lives in Asia, so a significant percentage of the world’s FSHD participants is expected to live there. To date, most FSHD studies have involved individuals of European descent, yet small-scale studies of East-Asian populations suggest that the likelihood of developing FSHD may vary. Here, we present the first genetically confirmed FSHD cohort of Indian ancestry, which suggests a pathogenic FSHD1 allele size distribution intermediate between European and North-East Asian populations and more asymptomatic carriers of 4 unit and 5 unit FSHD1 alleles than observed in European populations. Our data provides important evidence of differences relevant to clinical diagnostics and underscores the need for global FSHD participation in research and trial-ready Indian FSHD cohorts.

## Introduction

Facioscapulohumeral muscular dystrophy (FSHD) is the third most common dystrophy (after Duchenne and myotonic muscular dystrophies) [1]. Phenotype typically involves progressive, often asymmetric involvement of facial, scapular and humeral muscles, with symptoms onset in the second decade [2]. FSHD can be underdiagnosed due to heterogenous presentation and technically-challenging genetic testing. The disease is caused by aberrant expression of embryogenic transcription factor DUX4 in muscle. The *DUX4* gene is embedded in the D4Z4 macrosatellite repeat array on chromosome 4. Typically, the polymorphic D4Z4 repeat array is 8-100 units (U) in size, and *DUX4* expression is repressed in most somatic tissues. In FSHD1, sustained *DUX4* expression in skeletal muscle results from repeat array contraction to 1-10U within a permissive haplotype (4qA) of chromosome 4, and resulting local chromatin relaxation [3]. The permissive 4qA haplotype contains a somatic polyadenylation signal stabilising the *DUX4* transcript (absent from non-permissive haplotype 4qB and also from a homologous region on chromosome 10). The 4qA allele is present globally (79% in Nigerian controls, 45% in European controls and 40% in Japanese and Chinese controls) [4, 5] and is subdivided into haplotypes to inform population genetic evaluation. The most common 4qA haplotype is 4A161, further divided into 4A161S and 4A161L subtypes based on the size (small [S] or long [L]) of the distal-most partial D4Z4 unit in the repeat array. Approximately 8% of European genomes contain the 4A161L haplotype, but this is absent from non-European population data [6]. In addition to repeat array contraction, 4qA-associated *DUX4* can be derepressed by D4Z4 chromatin relaxation on chromosomes 4 and 10 (measured by D4Z4 hypomethylation). Here, pathogenic variants in chromatin modifiers, most often in Structural Maintenance of Chromosomes flexible Hinge Domain Containing 1 (SMCHD1), are involved, and the disease is classified as FSHD2 [7–9].

FSHD severity correlates with repeat array size: 1-3U FSHD1 participants display greatest chromatin relaxation, more severe phenotype and earlier onset than people with 8-10U FSHD1 alleles [10, 11]. However, even after age-correction, FSHD shows high clinical variability between individuals of identical FSHD1 allele size within and between families. This is especially true of 8-10U repeat arrays, which also occur in 1–2% of the unaffected European population: multiple epigenetic factors may influence variability [12].

The gold standard for FSHD diagnostics involves sequential digests and Southern blotting, requiring technical proficiency and quality checks, however, comparable results are now possible with molecular combing and genome optical mapping approaches [13–18]. All require very high molecular weight DNA.

At the time of writing, Indian FSHD diagnostics is predominantly clinical: in-country genetic testing has recently been established, but cost can impact access. FSHD genetic research has been performed primarily on people from Europe, North America and Northeast Asia (Japan, Korea and China). Previous studies revealed the FSHD1-allele size range in participants with a Northeast Asian genetic background is generally shorter (1-6U) than in participants with a European genetic background (1-10U), suggesting higher FSHD susceptibility in European populations [19–21]. This apparent population-based variability in severity challenges the use of current European thresholds for FSHD1 in non-European populations, including the understudied Indian population.

## Materials and methods

### Participant recruitment

Participants referred to the All India Institute of Medical Sciences (AIIMS) New Delhi Neurology and Comprehensive Neuromuscular Disorders Clinic (AIIMS-CNMD) provided informed consent to participate in the International Centre for Genomic Medicine in Neuromuscular Diseases (ICGNMD) study for phenotypic, clinical and family history data collection and research testing [22].

### Inclusion and exclusion criteria

Inclusion and exclusion criteria were based on an existing FSHD physician diagnostic criteria (https://www.urmc.rochester.edu/MediaLibraries/URMCMedia/neurology/documents/Physician-checklist-FSHD-Final-clean.pdf) to maximise recruitment of a range of FSHD phenotypic severities (mild to severe) while minimising recruitment of participants more likely to have other diseases with similar phenotypes.

Inclusion criteria for probands (index cases):

1. Weakness of facial muscles and/or
2. Weakness of scapular stabilisers and/or foot dorsiflexors.

Exclusion criteria

1. Presence of ptosis or weakness of extraocular muscles and/or
2. Muscle biopsy (where available) in participant or affected relative with features suggesting an alternative diagnosis and/or
3. EMG in participant or affected relative showing myotonia or neurogenic changes.

**Fig. 1:**
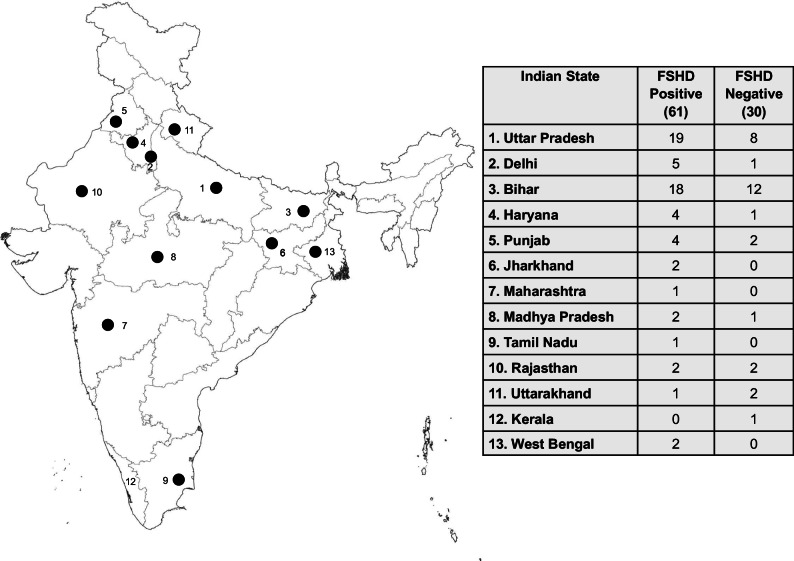
Map marking Indian state of birth of the 91 probands, with numbers of genetically confirmed FSHD positive or negative probands recruited shown in the table. Number on map: State Code in Table; Black circle: FSHD positive case(s) in state.

We screened 1165 participants and recruited 91 probands and 144 available family members (235 individuals, of which 155 (66%) male). To maximise the range of FSHD severities recruited, we invited all close relatives to participate in the study, regardless of phenotypic status. Participants’ state of birth was collected (Fig. 1).

All participants were examined by the same neurologist (VVY) who documented clinical evaluation scores (Ricci clinical severity score (CSS), age-corrected clinical severity score (ACSS = [(CSS*2)/AAE]*1000, where AAE = age at examination), FSHD Clinical Score and age-corrected FSHD Clinical Score ( = [(FSHD Clinical Score)/AEE]*1000), and Medical Research Council (MRC) sum-score) [23–25]. Participants were investigated for muscle disease (Creatine kinase, electromyography, Muscle MRI). We defined genetically confirmed FSHD participants as carriers of 1-10U permissive 4qA allele (FSHD1) and/or carriers of permissive 4qA allele and D4Z4 CpG hypomethylation on chromosomes 4 and 10 below FSHD2 threshold (FSHD2). We also analysed FSHD2 participants for pathogenic variants in relevant genes to confirm FSHD2. We applied standard classification of genetically FSHD participants [2]. “Symptomatic” participants had muscle symptoms on history and muscle FSHD signs on examination (equivalent to one or both inclusion criteria). “Asymptomatic” participants did not report symptoms of FSHD on history-taking; however, the examination did find muscle signs of FSHD present (typically mild signs). “Nonpenetrant carrier” participants were “genetically FSHD” but had no history of muscle symptoms and also had no muscle FSHD signs on examination.

### FSHD genetic testing strategy

We applied a stepwise FSHD genetic testing strategy (Supplementary Fig. 1). First, FSHD1 genetic analysis was done by Southern blotting and PCR analysis in all probands (*N* = 91). As sites globally are introducing FSHD optical genome mapping (OGM), we also tested thirteen probands with this to confirm concordance. If probands (by definition symptomatic) were genetically FSHD1 (1-10U allele size), we expanded FSHD1 analysis to identify symptomatic, asymptomatic and nonpenetrant genetically FSHD1 relatives. Probands genetically negative for FSHD1 (>10U allele size) and with at least one permissive allele were next analysed for FSHD2 (*N* = 24), as were genetically FSHD1 participants carrying 7-9U FSHD1 alleles (*N* = 8), and *in cis* duplication alleles (*N* = 2). Relatives of the five confirmed FSHD2 probands were also tested (*N* = 10), bringing the total methylation tests to 44. Upon detecting FSHD2 based on methylation testing, positive probands (*N* = 5) had whole exome sequencing to identify FSHD2-related genetic variants. Finally, genetically FSHD-negative probands (*N* = 30) also had whole exome sequencing and were transferred to a larger, ongoing “unsolved” ICGNMD neuromuscular disease cohort for further analysis. In parallel to our study, seven FSHD-negative participants also obtained a dystrophin duplication/deletion test.

### FSHD genetic testing protocols

EDTA blood samples were stored for 1 to 8 weeks at 4 degrees Celsius before testing. All shipped samples were insulated in polystyrene to maintain cool temperatures and avoid freezing in transit. Southern blot was undertaken at Leiden University Medical Centre (The Netherlands) and the All India Institute of Medical Sciences (India), with isolated white blood cells embedded in agarose plugs to obtain high molecular weight DNA as previously described [18]. DNA in agarose underwent restriction enzyme digest followed by pulsed-field gel electrophoresis (PFGE), Southern blotting and sequential hybridisation with radio-labelled probes for p13E-11, D4Z4, 4qA and 4qB. Finally, haplotype analysis was completed via SSLP PCR as described [18]. PCR haplotyping for 4qA-S and 4qA-L was done as described [6]. For all participants with >7U D4Z4 repeat array on chromosome 4qA, we determined methylation at the FseI site in D4Z4 on chromosomes 4 and 10 and calculated delta1 methylation score to identify FSHD2 and to reveal differences in CpG methylation at D4Z4 that could correlate with clinical variability in FSHD1 [11, 18]. Optical genome mapping was performed at University College London (UK) on DLS-labelled HMW DNA from 13 probands using the Saphyr Genome Imaging Instrument (1-colour) following manufacturer’s guidelines and as described [26] (https://bionano.com/support-documentation/).

Whole exome sequencing (WES) was performed at Macrogen Europe (Amsterdam) to check for FSHD2 genetic variants, classified using ACMG criteria [27].

Duchenne or Becker Muscular Dystrophy DMD multiplex ligation-dependent probe amplification (MLPA) results were obtained from LifeCell Diagnostics (Chennai) using MRC Holland P034 DMD-1 and P035 DMD-2 SALSA MLPA probe mixes, according to manufacturer protocols.

### Statistical analysis of severity

Pearson (parametric) and Spearman’s Rho (non-parametric) correlations were performed to assess the correlation between disease severity and repeat size using IBM_SPSSS_27.

## Results

### FSHD1 genetic analysis

**Fig. 2:**
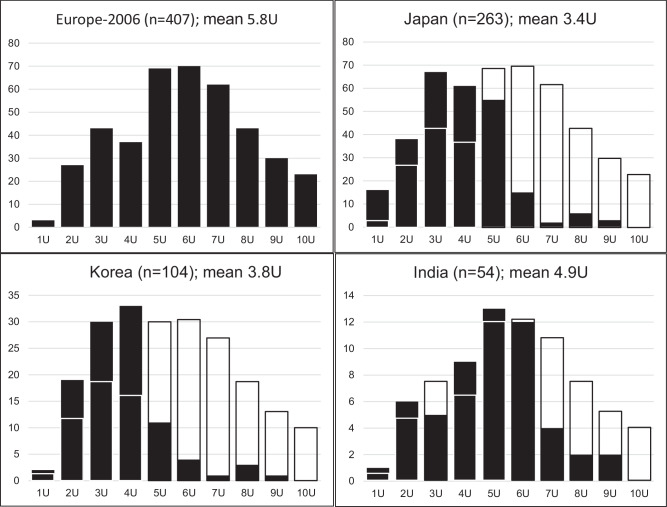
Comparison of D4Z4 repeat array size distribution of FSHD1 alleles in unrelated FSHD1 patients from India (this study) and in other populations based on previous publications [19–21]. Plot titles show number of FSHD alleles (in parentheses), and mean repeat array size. *X* axes: repeat array size in units (U) and *Y* axes: D4Z4 repeat array size. For non-European populations, European data is underlaid in outline (*Y* axis not to scale) to illustrate the latter’s relatively broad allelic range.

FSHD1 genetic variability in India has not been evaluated specifically before, so we first assessed genetic and clinical data with reference to observed threshold differences between European and Northeast Asian populations. Southern blot-based genotype analysis revealed a 1-9U 4qA allele (Europe-based FSHD1 threshold) in 57 of 91 families. In 5 of the 57 families (probands IC_AIM_000788, IC_AIM_000817, IC_AIM_000828, IC_AIM_000932, IC_AIM_01146), we identified a 9U-sized 4qA allele (no 10U found). As 9U is outside the FSHD1 range in Northeast Asian participants, we analysed these families in more detail. In two families, the probands (IC_AIM_000817, IC_AIM_000932) were also FSHD2, with D4Z4 hypomethylation levels below the FSHD2 threshold and a pathogenic variant in SMCHD1. The father of one of these probands shared the 9U 4qA allele but not the pathogenic SMCHD1 variant and was unaffected. A third 9U proband (IC_AIM_00788) had an in-frame deletion of exon 45–48 in Dystrophin, suggesting Becker Muscular Dystrophy (BMD), which in retrospect better fitted clinical features. This 14 year-old was initially included due to mild bifacial weakness, asymmetry in proximal lower limb weakness and scapular winging. The clinical pointer to BMD was calf hypertrophy and moderately raised CK (2234). The fourth proband (IC_AIM_01146) was homozygous for the 9U 4qA allele, with two heterozygous, non-penetrant parents. FSHD1 is a dominant hereditable disease in which homozygosity is rarely observed but is known to result in phenotypic dosage [28]. The fifth 9U 4qA allele proband (IC_AIM_00828) did not have D4Z4 hypomethylation levels suggestive of FSHD2 and showed facial weakness, scapular winging and asymmetric proximal upper limb weakness. The relatively severe phenotype in relation to FSHD1 allele size (CSS 1.5, age-corrected CSS 125, FSHD Clinical score 3) suggests other contributing factors; however, whole exome sequencing identified no candidates. After excluding the BMD and FSHD2 participants, 54/91 (59%) probands were genetically FSHD1, and 100 individuals from 55 families were genetically FSHD1 (54 FSHD1 probands and 46 relatives). Relatives included two participants with a different allele size to their proband; one was an unaffected 8U 4qA allele carrier and sibling of a 4U FSHD1 proband, and the other was a 9U 4qA carrier without hypomethylation from an FSHD2 family. Overall, FSHD1 allele size distribution more closely resembled the distribution observed in Japanese and Korean populations than in European populations (Fig. 2).

### Haplotype distribution

To better understand FSHD haplotypes within the Indian population, we analysed the frequency of permissive (4qA) and non-permissive (4qB) haplotypes. To estimate haplotype frequency in the healthy Indian population, we included all unique alleles within each family in our cohort except those directly contributing to FSHD. We also excluded healthy parental 4qA alleles contracted in the subsequent generation in de novo FSHD1 probands. We added data from Gujarati Indians in the International HapMap Project (https://www.genome.gov/10001688/international-hapmap-project). Overall, we found a 4qA permissive haplotype frequency in the Indian control population of 46% (182 alleles), comparable to European population frequency (45%). The globally most common permissive haplotype (4A161) comprised 158/182 of the Indian 4qA alleles (87%). Five were of the 4A161L haplotype (1%), previously observed only in European populations (8%) [6] and absent in HapMap individuals from Northeast Asia and Sub-Saharan Africa (Supplementary Table 1).

### De novo versus inherited FSHD1

**Fig. 3:**
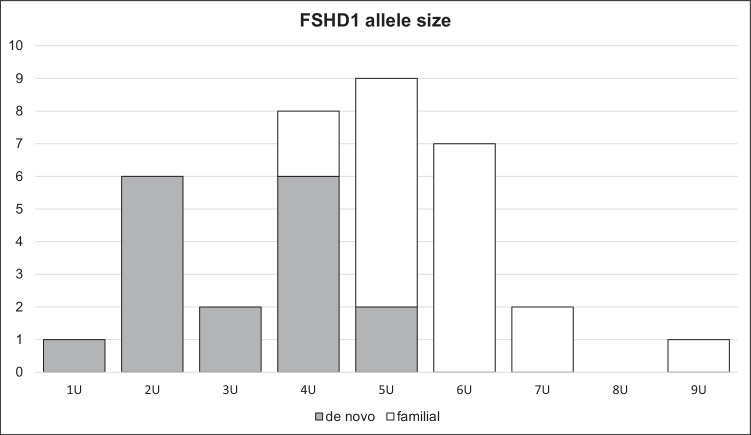
Repeat array size distribution in de novo versus familial FSHD. FSHD1 D4Z4 repeat array size distribution for families where we could confirm that the allele originated from a new contraction (de novo) and where the FSHD1 allele was inherited from an earlier generation (familial).

Analysis of the 54 confirmed FSHD1 probands (Fig. 2) and recruited family members showed a de novo D4Z4 repeat array contraction in 17 families: nine were germline mutations, and eight resulted in somatic mosaicism (where only a percentage of cells are affected). For the other 37 probands, 19 showed familial inheritance with genetically confirmed FSHD1 carriers, while 18 probands had no family members available to assess inheritance. Based on available information, at least one-third have de novo mutations. As shown in previous studies [10], FSHD allele size in FSHD-affected families is generally larger than in participants with de novo mutations (Fig. 3).

### FSHD2 genetic analysis

We identified 5 FSHD2 probands (IC_AIM_000817, IC_AIM_000932, IC_AIM_01192, IC_AIM_01162 and IC_AIM_01042). These participants had a permissive allele of 9-19U (usually 8-20U), an additive effect of a second permissive allele in this size range, a delta1 methylation between −27% and −41% (FSHD2 threshold <−20%) and a pathogenic or likely pathogenic variant in SMCHD1 (NM_015295.3:c.823A>G p.(Lys275Glu), c.1056C>G p.(Tyr352*), c.1580C>T p.(Thr527Met), c.3051_3075del p.(Ser1017Argfs*20) and c.4101del p.(Lys1367Asnfs*16) (Supplementary Table 2).

### FSHD with dominant *in cis* duplication allele

Two families (probands IC_AIM_00152 and IC_AIM_00875) carried a 4qA allele with an *in cis* duplication of the D4Z4 array and control levels of methylation, leading to classification as autosomal dominant *in-cis* duplication FSHD [29–31]. The parents of IC_AIM_00875 were recruited, with data suggesting a proband de novo duplication. The duplication allele in IC_AIM_00152 was also confirmed by OGM [16].

Overall, FSHD was confirmed in 61 (67%) of the 91 families (54 (89%) FSHD1, five (8%) FSHD2 and two (3%) with autosomal dominant *in cis* duplication alleles. The “confirmed FSHD” percentage was comparable to those in European and Northeast Asian studies. Thirteen probands were additionally analysed by OGM: results agreed with PFGE-based Southern blot analysis (for example, see Supplementary Fig. 1).

### FSHD clinical characteristics

Summary data for all non-mosaic genetically FSHD participants was collated (Table 1A–C). Eight additional mosaic FSHD1 participants, whose severity is influenced by the fraction of affected cells in combination with residual D4Z4 repeat array size [32], were evaluated separately. The FSHD1 group (Table 1A) included 66 symptomatic participants, 26 asymptomatic carriers and eight nonpenetrant carriers. The majority were male (61/92; 66%) in their second or third decades (median age 23 years (range 9–57)). There was a median delay of 10 years between the onset of symptoms (median 16, range 0–37) and clinical diagnosis (median 26, range 9–55). Median CK was 417 U/L (range 20–1288). Muscle biopsy for 7 participants suggested dystrophy, but with normal immunostaining for dystrophin, dysferlin, sarcoglycan and calpain (as befits exclusion criteria). The EMG of all probands was myopathic. The most common first symptoms were proximal upper limb muscle weakness (41/92; 45%) and facial muscle weakness (9/92; 10%). The presenting symptom of proximal muscle weakness was seen more in males than females (57% versus 19%). The most common clinical features at diagnosis were moderate to severe facial weakness (83/92; 90%), scapular winging (76/92; 83%), and proximal upper limb weakness (64/92; 70%). No participant reported vision, hearing, or breathing abnormalities. Most symptomatic and asymptomatic participants had asymmetric muscle weakness on examination (70/92; 76%). Beevor’s sign was seen in 42/92 (46%) participants, more commonly in males (35/61; 57%) than females (7/31; 23%). Asymmetric quadriceps weakness was seen in 10/92 (11%) participants, and shoulder pain was present in 39/92 (42%) participants. Ambulation was normal in 40/92 (40%) participants. No participant had lost ambulation (i.e., none were always wheelchair-bound). Around two-thirds of symptomatic participants had symptoms onset before 18 years (50/71; 70%). Ten participants (17/71; 24%) had symptoms onset before 12 years. Positive family history of symptoms was confirmed or reported in 45/91 (50%) families (38 families had genetically confirmed relatives, but seven families’ affected relatives were unavailable for testing).

**Table 1:** Clinical and laboratory features of genetically-confirmed FSHD participants (probands and relatives), showing male, female and combined sex data.

	A. Genetically confirmed FSHD1 (symptomatic, asymptomatic and nonpenetrant) (*N* = 92)			B. Genetically confirmed FSHD2 (symptomatic, asymptomatic and nonpenetrant) (*N* = 7)			C. Genetically confirmed *in cis* duplication FSHD (*N* = 2)		
	Female	Male	Overall	Female	Male	Overall	Female	Male	Overall
Total	31 (34%)	61 (66%)	92	1 (14%)	6 (86%)	7	1 (50%)	1 (50%)	2
Proband	9	43	52	0	5	5	1	1	2
Affected relatives	22	18	40	1	1	2	0	0	0
Age at examination (yrs): median (range)	28 (9–55)	22 (11–57)	23 (9–57)	35	21.5 (18–60)	24 (18–60)	27	20	23.5 (20,27)
Consanguineous marriage	0	4	4/92 (4%)	0	0	0	0	0	0
Family History	7 probands	25 probands	32/92 (34%)	0 probands	1 proband	1/7 (14%)	0	0	0
Age of onset of first symptom - median (range)	16 (3–51)	15 (0–37)	16 (0–51)	N/A	21.5 (10–52)	21.5 (10–52)	11	17	14 (11,17)
Age of Diagnosis; median (range)	28 (9–55)	22 (10–51)	25.5 (9–55)	35	21.5 (18–60)	24 (18–60)	27	20	23.5 (20–27)
Age of onset of diminished ability; median (range)	17 (8–51)	15 (7–37)	17 (7–51)	N/A	22 (12–55)	22 (12–55)	20	17	18.5(17,20)
First symptom	Female	Male	Overall	Female	Male	Overall	Female	Male	Overall
Facial muscle weakness	5/31 (16%)	4/61 (7%)	9/92 (10%)	0	1/6 (17%)	1/7 (14%)	0	0	0
Proximal muscle weakness in upper limbs	6/31 (19%)	35/61 (57%)	41/92 (45%)	0	4/6 (67%)	4/7 (57%)	1/1 (100%)	0	1/2 (50%)
Proximal muscle weakness in lower limbs	3/31 (10%)	4/61 (7%)	7/92 (8%)	0	0	0	0	1/1 (100%)	1/2 (50%)
Scapular winging	0	2/61 (3%)	2/92 (2%)	0	0	0	0	0	0
Distal weakness in upper limb	0	0	0	0	0	0	0	0	0
Foot Dorsiflexor weakness	0	3/61 (5%)	3/92 (3%)	0	0	0	0	0	0
Hearing/Vision abnormality	0	0	0	0	0	0	0	0	0
Developmental delay	0	1/61 (2%)	1/92 (1%)	0	0	0	0	0	0
Asymptomatic/Non penetrant	17/31 (55%)	12/61 (20%)	29/92 (32%)	1	1/6 (17%)	2/7 (29%)	0	0	0
Clinical features	Female	Male	Overall	Female	Male	Overall	Female	Male	Overall
Facial muscle weakness	25/31 (81%)	58/61 (95%)	83/92 (90%)	0	5/6 (83%)	5/7 (71%)	1/1 (100%)	0	1/2 (50%)
Proximal muscle weakness in upper limbs	17/31 (55%)	47/61 (77%)	64/92 (70%)	0	6/6 (100%)	6/7 (86%)	1/1 (100%)	1/1 (100%)	2/2 (100%)
Proximal muscle weakness in lower limbs	8/31 (25%)	26/61 (43%)	34/92 (37%)	0	3/6 (50%)	3/7 (43%)	1/1 (100%)	1/1 (100%)	2/2 (100%)
Scapular winging	22/31 (71%)	54/61 (89%)	76/92 (83%)	0	6/6 (100%)	6/7 (86%)	1/1 (100%)	0	1/2 (50%)
Distal LL muscle weakness	3/31 (10%)	15/61 (25%)	18/92 (20%)	0	2/6 (33%)	2/7 (29%)	0	0	0
Distal UL muscle weakness	1/31 (3%)	12/61 (20%)	13/92 (14%)	0	2/6 (33%)	2/7 (29%)	0	0	0
Lordosis/Scoliosis	6/31 (19%)	25/61 (41%)	31/92 (34%)	0	3/6 (50%)	3/7 (43%)	0	0	0
Beevor	7/31 (23%)	35/61 (57%)	42/92 (46%)	0	5/6 (83%)	5/7 (71%)	0	1/1 (100%)	1/2 (50%)
Hearing/vision abnormality	0	1/61 (2%)	1/92 (1%)	0	0	0	0	0	0
Quadriceps muscle weakness	1/31 (3%)	9/61 (15%)	10/92 (11%)	0	0	0	0	0	0
Shoulder pain	10/31 (32%)	29/61 (48%)	39/92 (42%)	0	5/6 (83%)	5/7 (71%)	1/1 (100%)	0	1/2 (50%)
Diabetes Mellitus	0	1/61 (2%)	1/92 (1%)	0	0	0	0	0	0
Distal Hyperlaxity	1/31 (3%)	2/61 (3%)	3/92 (3%)	0	0	0	0	0	0
Pectus excavatum	0	4/61 (7%)	4/92 (4%)	0	0	0	0	0	0
Popeye arm	0	11/61 (18%)	11/92 (12%)	0	0	0	0	0	0
Other neurological and systemic involvement	0	1/61 (2%)	1/92 (1%)	0	0	0	0	0	0
Muscle Biopsy	3/31 (10%)	4/61 (7%)	7/92 (8%)	0	0	0	O	1/1 (100%)	1/2 (50%)
Muscle MRI	6/31 (19%)	13/61 (21%)	19/92 (21%)	0	3/6 (50%)	3/7 (43%)	0	0	0
CK U/L	198 (20–418)	486 (46–1288)	417 (20–1288)	N/A	417 (209–1214)	417 (209–1214)	519	2890	1705 (519,2890)
CK 500–1000 U/L	0	14/61 (23%)	14/92 (15%)	0	0	0	1/1 (100%)	0	1/2 (50%)
CK > 1000 U/L	0	4/61 (7%)	4/92 (4%)	0	1/6 (17%)	1/7 (14%)	0	1/1 (100%)	1/2 (50%)
Severity Scores: Median (range)	Female	Male	Overall	Female	Male	Overall	Female	Male	Overall
Total MRC sum score (0–70)	68.5 (48–70)	60.5 (43–70)	62 (43–70)	70	58 (47–62)	59 (47–70)	54	52	53 (52,54)
Clinical severity Scale (CSS; 0–5)	1.5 (0–4)	2 (0–4.5)	2 (0–4.5)	0	0.5 (1.5–4.5)	0.5 (0–4.5)	3	3	3
Age corrected CSS	118 (0–800)	167 (0–538)	163 (0–800)	0	216 (58–421)	216 (0–421)	222	222	222
FSHD clinical score (0–15)	3 (0–12)	7 (3–13)	5.5 (0–13)	0	7.5 (5–13)	7.5 (0–13)	7	7	7

Eight mosaic participants are excluded from comparisons due to variable penetrance impacting clinical measures (see text). 1 A summarises FSHD1 participants, 1B FSHD2 participants and 1 C *in cis* duplication participants.

*N/A* not applicable.

The genetically FSHD2 group (Table 1B) included seven participants (five symptomatic male probands, one asymptomatic relative and one non-penetrant relative). The clinical features were indistinguishable from FSHD1 participants. An autosomal dominant *in cis* duplication allele was present in two unrelated symptomatic probands (Table 1C). These also were similar clinically to FSHD1, although both participants had relatively elevated CK (519 and 2890 U/L).

The clinical severity of FSHD1 roughly  correlates with the size of repeat array contraction. We classified genetically-confirmed FSHD1 symptomatic and asymptomatic participants as having 1-3U or 4-9U (Table 2). Age of onset was younger (median, 10 years) for the 1-3U FSHD1 allele group (Table 2A) compared to the 4-9U group (median, 16 years; Table 2B), and age-corrected CSS score was also higher (median 429 (range 130–538) versus median 200 (range 29–500)). All but one 1-3U participants were recruited as probands (i.e., had clinical features warranting referral), and so high incidences of facial weakness and scapular or foot dorsiflexor weakness were expected: these were not required for relatives’ study recruitment, who represented 49% (39/80) of the 4-9U group.

**Table 2:** Comparison of clinical and laboratory features of FSHD1 participants carrying 11-3 D4Z4 repeat units versus 4-7 repeat units.

	A. Genetically confirmed FSHD1 probands, excluding mosaic probands (*N* = 52)		B. Probands and Relatives with genetically-confirmed FSHD1 (1-9U), excluding mosaic carriers (*N* = 92)	
	1-3U	4-9U	1-3U	4-9U
Proband	11/11	41/41	11/12 (92%)	41/80 (51%)
Affected relative	-	-	1/12 (8%)	39/80 (49%)
Sex: Male (M), Female (F), *N* (%)	F = 3/11 (27%) M = 8/11 (73%)	F = 6/41 (15%) M = 35/41 (85%)	F = 3/12 (25%) M = 9/12 (75%)	F = 28/80 (35%) M = 52/80 (65%)
Age at examination (yrs): median (range)	15 (10–29)	22 (15–57)	18.5 (10–50)	24 (9–57)
Consanguineous marriage	1/11 (9%)	2/41 (5%)	1/12 (8%)	5/80 (6%)
Family History	5/11 (45%)	24/41 (58%)	6/12 (50%)	57/80 (71%)
Age of onset of first symptom	10 (3–22)	16 (7–51)	10 (3–22)	16 (7–51)
Age of Diagnosis	15 (10–28)	22 (13–55)	18.5 (10–50)	23 (9–55)
Age of onset of diminished ability	10 (7–22)	18 (8–51)	10 (7–22)	18 (8–51)
First ever symptom	1-3U	4-9U	1-3U	4-9U
Facial muscle weakness	6/11 (55%)	2/41 (5%)	6/12 (50%)	3/80 (4%)
Proximal muscle weakness in upper limb	4/11 (36%)	30/41 (73%)	5/12 (42%)	36/80 (45%)
Proximal muscle weakness in lower limb	0	9/41 (22%)	0	9/80 (11%)
Scapular winging	0	1/41 (2%)	0	2/80 (3%)
Foot dorsiflexor weakness	0	0	0	1/80 (1%)
Asymptomatic	0	12/41 (29%)	0	24/80 (30%)
Clinical feature	1-3U	4-9U	1-3U	4-9U
Facial muscle weakness	8/11 (73%)	38/41 (93%)	9/12 (75%)	69/80 (86%)
Proximal muscle weakness in upper limb	10/11 (91%)	33/41 (80%)	11/12 (92%)	47/80 (59%)
Proximal muscle weakness in lower limb	8/11 (73%)	23/41 (56%)	8/12 (67%)	27/80 (34%)
Scapular winging	9/11 (82%)	38/41 (93%)	10/12 (83%)	64/80 (80%)
Distal LL muscle weakness	2/11 (18%)	8/41 (20%)	3/12 (25%)	10/80 (13%)
Distal UL muscle weakness	3/11 (27%)	3/41 (7%)	3/12 (25%)	3/80 (4%)
Lordosis/Scoliosis	8/11 (73%)	15/41 (37%)	9/12 (75%)	15/80 (19%)
Beevor sign	9/11 (82%)	22/41 (54%)	10/12 (83%)	28/80 (35%)
Hearing/vision abnormality	0	0	1/12 (8%)	0
Selective Quadriceps weakness	5/11 (45%)	3/41 (7%)	5/12 (42%)	3/80 (4%)
Shoulder pain	10/11 (91%)	13/41 (32%)	11/12 (92%)	21/80 (26%)
Diabetes Mellitus	0	1/41 (2%)	0	1/80 (1%)
Distal Hyperlaxity	1/11 (9%)	1/41 (2%)	1/12 (8%)	1/80 (1%)
Pectus excavatum	0	3/41 (7%)	0	3/80 (4%)
Muscle Biopsy	0	6/41 (15%)	0	6/80 (8%)
Muscle MRI	5/11 (45%)	13/41 (32%)	5/12 (42%)	14/80 (18%)
CK U/L	437 (20–1007)	276 (88–924)	437 (20–1007)	407 (46–1288)
CK 500–1000	3/11 (27%)	1/41 (2%)	3/12 (25%)	11/80 (14%)
CK > 1000	1/11 (9%)	0	1/12 (8%)	0
Severity Scores: Median (range)	1-3U	4-9U	1-3U	4-9U
Total MRC sum score (0–70)	58 (48–63)	59 (46–69)	58 (48–63)	62 (43–70)
Clinical severity Scale (CSS;0–5)	3.5 (1.5–4)	1.5 (0.5–4)	3.5 (1.5–4)	3 (0.5–4.5)
Age corrected CSS	428.5 (130.4–538.4)	200 (28.5–500)	396.1 (130.4–538.4)	142.8 (0–500)
FSHD clinical score (0–15)	9 (5–13)	6 (1–12)	9 (5–13)	5 (0–12)

Eight mosaic participants are excluded from comparisons due to variable penetrance impacting clinical measures (see text). 2A: data for probands only, 2B: combined data for probands and relatives.

*N/A* not applicable.

**Fig. 4:**
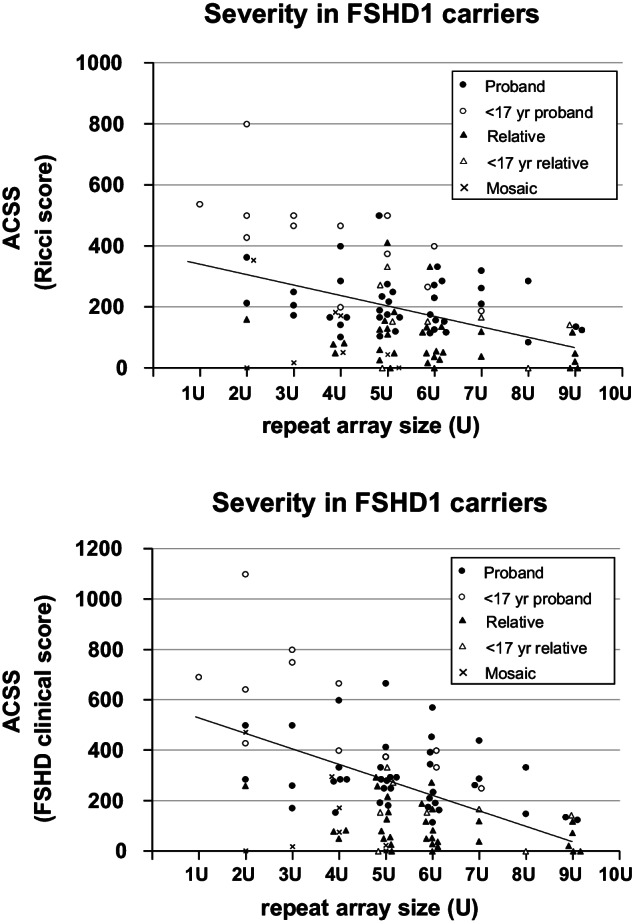
Graph showing age corrected clinical severity scores based on age adjusted Ricci scale (top) and age adjusted FSHD clinical score (bottom) for all participants in our cohort that carry an FSHD1-sized 4qA allele (*N* = 100). Adult proband: black circle, Proband under 17 yr: white circle, Adult relative: black triangle, Relative under 17 yrs: white triangle, Mosaic participant: x symbol. The regression line is based on all non-mosaic participants (*N* = 92). Y axes; Clinical Score, X axis; repeat array size.

We assessed the statistical correlation between FSHD1 severity and repeat size for all non-mosaic, genetically FSHD1 participants (*N* = 92). As expected, there was a highly significant inverse moderate correlation between repeat length and disease severity for both the Ricci age-corrected Clinical Score (ACCS) (R = −0.491, *p* < 0.001) and the age-corrected FSHD Clinical Score (R = −0.532, *p* < 0.001) (Fig. 4). Each reduction of repeat length correlated with an increase in the ACSS of 41 points and in the age-corrected FSHD Clinical Score of 61 points. As expected, due to asymptomatic and non-penetrant relatives, probands had significantly higher clinical severity than family members for both ACCS (267 ± 146 vs 104 ± 98, *p* < 0.001) and age-corrected FSHD Clinical Score (364 ± 200 vs 109 ± 94, *p* < 0.001). When the effect of proband versus family member status was taken into consideration, there was no significant difference between male and female participants.

We assessed non-mosaic FSHD1 allele distribution within symptomatic, asymptomatic and non-penetrant participants. To avoid any possibility of bias caused by very young participants not manifesting symptoms, we excluded all asymptomatic and non-penetrant relatives under 17 years old and included only probands and relatives with non-mosaic FSHD1 that displayed variable familial penetrance. The resulting dataset included 27 symptomatic participants (including 18 probands, all >3U), 17 asymptomatic and four nonpenetrant participants (Supplementary Fig. 3). Six of 22 asymptomatic participants carried a 4 or 5U FSHD1 allele (27%), as did one nonpenetrant participant (4.5%). A similar study of 10 Dutch families identified a single asymptomatic participant (and no nonpenetrant) of 4-5U (4.5%) [2]. Across both cohorts, asymptomatic and nonpenetrant participants of 6U or above were common (India 10/21 (48%), The Netherlands 18/33 (54%)).

The eight, unrelated mosaic FSHD1 participants in our cohort (Table 3) included three symptomatic, three asymptomatic and two nonpenetrant participants. Symptomatic individuals’ clinical signs did not differ from non-mosaic participants.

**Table 3:** Characteristics of eight study participants with FSHD somatic mosaicism. N/A = not applicable.

Study ID	Recruitment Detail	Age at examination	Symptomatic/ Asymptomatic/ Nonpenetrant carrier	Clinical Features	Size (U) and fraction (%) mosaic FSHD1 and parental alleles	MRC Sum Score	Clinical Severity Score (CSS)(0–5)	Age corrected CSS	FSHD Clinical Score
IC_AIM_00701	Father of non-mosaic IC_AIM_00551	60	Nonpenetrant	Not applicable (N/A)	[2U(40%);20U(60%)]	70	N/A	N/A	N/A
IC_AIM_00224	Mother of non-mosaic IC_AIM_00222	45	Asymptomatic	Mild scapular winging	[5U(60%); 31U(40%)]	70	1	44	1
IC_AIM_00961	Father of non-mosaic IC_AIM_00904	58	Asymptomatic	Mild Facial weakness	[3U(50%);19U(50%)]	70	0.5	17	1
IC_AIM_00413	Proband	30	Symptomatic (age of onset = 20 yrs)	Proximal UL weakness (asymmetric) Proximal LL weakness (asymmetric) Bilateral foot drop Bifacial weakness Popeye arm	[4U(75%); 26U(25%)]	43	4	267	13
IC_AIM_00715	Mother of non-mosaic IC_AIM_00123	35	Symptomatic (age of onset = 20 yrs)	Bifacial weakness Proximal UL weakness Scapular winging Lumbar hyperlordosis Beevor Shoulder Pain	[4U(60%); 37U(40%)]	61	3	174	6
IC_AIM_01083	Proband	17	Symptomatic (age of onset = 12 yrs)	Weakness of facial musculature Proximal muscle weakness in upper limbs Scapular winging Distal lower limb muscle weakness Proximal muscle weakness in lower limbs Hyperlordosis Elbow flexion contracture Shoulder pain Beevor sign	[2U(10%);19U(90%)]	56	3	353	8
IC_AIM_01215	Father of non-mosaic IC_AIM_01179	55	Nonpenetrant	N/A	[5U(30%);16U(70%)]	70	N/A	N/A	N/A
IC_AIM_01403	Mother of non-mosaic IC_AIM_01401	40	Asymtomatic	Beevor sign Bifacial weakness Scapular winging	[4U(95%);19U(5%)]	70	1	50	3

### Genetically non-FSHD participants

We could not genetically confirm FSHD1 or FSHD2 in 30 probands (Supplementary Table 3). Twenty-four (80%) were male, and the median age of onset was 28 years. Common presenting symptoms included proximal lower limb weakness and upper limb weakness. Facial weakness was present in 27 (90%) probands (mild in 25). Asymmetry was present in 18 (60%) probands. One participant had Beevor’s sign, and one was wheelchair-bound. Seven participants had CK > 1000 U/L (23%), and seven had muscle biopsy suggestive of muscular dystrophy. One female proband (IC_AIM_00786; 28 years, CK 2358, age of onset 19 years) was subsequently diagnosed as a manifesting carrier of DMD (confirmed by biopsy and MLPA showing heterozygous out-of-frame deletion of exons 45–50). She had asymmetric proximal upper limb weakness, proximal lower leg weakness, scapular winging, lordosis and mild facial weakness. (Another male proband with BMD (IC_AIM_00788) is discussed separately due to their 9U FSHD1 repeat size, nevertheless, we do consider this another BMD diagnosis, not FSHD1). Three other participants had possible other causes identified by whole exome sequencing (IC_AIM_00713; homozygous DYSF NM_003494.4:c.1020C>A p.(Ser340Arg), IC_AIM_00804; homozygous CAPN3 NM_000070.3:c.2050+2T>C, and IC_AIM_00478 homozygous TRIM32 NM_012210.4:c.357dup p.(Cys120Metfs*2). All FHSD-negative participants will undergo future analysis as an “unsolved” neuromuscular cohort.

## Discussion

There is currently a bias in inherited disease cohorts towards predominantly European ancestry. This is important to address as people of non-European ancestries may be at increasing risk of false positive or false negative diagnoses as genetic diagnostic testing expands globally. An accurate genetic diagnosis is important for FSHD, which is challenging to diagnose clinically and to differentiate from other conditions; based on this and previous studies, only around half of clinically suspected FSHD participants are genetically confirmed after testing [33, 34]. Previous studies show FSHD exhibits regional variation: cohorts from Japan and South Korea (here referred to as “Northeast Asian populations”) have generally shorter FSHD1 alleles in FSHD positive participants than European populations (1-6U, versus 1-10U) [19–21]. This has diagnostic implications, as relatives carrying a 7-10U 4qA allele may have a greater or lesser risk of developing FSHD symptoms, depending on ancestry.

We describe the first large genetically confirmed FSHD cohort from India. Due to AIIMS’ location in Delhi, most participants are from northern India; expanding southern populations in future studies will further improve knowledge of India’s diversity. On-going initiatives to connect clinicians, diagnostic centres of excellence and participant-led organisations to collate data across India will be important to increase knowledge.

Our study’s inclusion criteria meant the majority of probands displayed weak facial muscles, weakness of scapular stabilisers or foot dorsiflexors. Moderate to severe facial weakness was seen in almost all genetically confirmed probands, while most FSHD-negative probands had mild facial weakness. No genetically-confirmed FSHD1 participant used a wheelchair all the time, and none had extra-muscular manifestations. The combined typical phenotype of facial weakness, proximal upper limb weakness and scapular winging was observed most often in carriers of a 1-3U FSHD1 allele. Half the cohort displayed positive Beevor’s signs. Two unusual features in our FSHD cohort were selective quadriceps weakness and shoulder pain. Shoulder pain was more common with 1-3U FSHD1 (11/12; 92%) than 4-9U (21/80; 26%). Early FSHD studies suggested sex-related severity differences not reported in later studies: the non-significant differences we observed may result from the higher proportion of male probands; probands have significantly higher severity than genetically FSHD non-probands. Greater male patient referral and clinic attendance are also reported by AIIMS, so severely affected female FSHD participants may have been relatively less accessible to our study [35]. Future, larger datasets would facilitate further analysis.

After testing 91 families (235 individuals) with clinically-suspected FSHD, based on standard clinical inclusion and exclusion criteria, we identified FSHD1 (1-9U) in 54 families (59%). Strikingly, an 8-10U FSHD1 allele was present in only four families (7.4%), compared to >17% of FSHD1 participants in studies with predominantly European ancestry [21]. The FSHD1 allele size distribution in our cohort seems intermediate between populations of Northeastern Asian ancestry and European ancestry. The presence of some 4A161L alleles in the Indian population also suggests an intermediate picture: this haplotype represents 8.3% of all European 4q haplotypes but is absent in Northeast Asian populations. While 8-10U FSHD1 can be observed in India, our results suggest individuals of European genetic ancestry with this allele size range are more susceptible to FSHD than individuals of Indian ancestry. Familial analysis supports this: across non-mosaic FSHD1 allele relatives of 18 probands, we identified 27 symptomatic, 17 asymptomatic and 4 nonpenetrant participants. Remarkably, six asymptomatic and one nonpenetrant participant carry 4U or 5U allele, a size usually pathogenic in European populations.

Our FSHD1 cohort included both de novo and familial participants. New FSHD1 mutations can occur in the germline or in early embryogenesis, resulting in gonosomal FSHD1 allele mosaicism. As expected, de novo FSHD participants had very short D4Z4 arrays (2-5U) and severe symptoms; familial FSHD1 alleles were generally longer, with milder symptoms. We observed a higher proportion of de novo participants in our cohort (17/54; 31%) compared to Europe (10–20%); a larger cohort is needed to confirm this before reasons can be considered [19–21, 33].

Five of the 91 families studied were found to have FSHD2 caused by an SMCHD1 variant. One known pathogenic missense variant NM_015295.2:c.1580 C>T p.(Thr527Met) is reported in four other FSHD2 families internationally (https://databases.lovd.nl/shared/genes/SMCHD1) [36, 37]. The other four variants are not previously described; three are predicted “stop gain” mutations c.1056C>G p.(Tyr352*), c.3051_3075del p.(Ser1017ArgfsTer20), and c.4101del p.(Lys1367Asnfs*16) and one (c.823A>G p.(Lys275Glu)) is an ATPase domain missense variant absent from gnomAD (gnomAD_v4.0.0_ENSG00000101596_2024_01_02_20_35_14). FSHD2 population frequency (ratio of FSHD2 to FSHD1 and FSHD2) is 5/61 (8%) in our cohort. Two recent studies showed lower (3-6%) FSHD2 frequency in the European population [17, 33]. While larger Indian datasets are needed, a higher proportion of FSHD2 in India might be expected if FSHD1 is less likely to develop with 8-10U.

Notably, two participants have FSHD caused by an *in cis* duplication allele. *In cis* duplication alleles were originally reported causative in FSHD2 participants with a pathogenic variant in SMCHD1 and D4Z4 hypomethylation [29], however our participants had D4Z4 methylation levels excluding FSHD2. Two recent studies report 15 FSHD families with autosomal dominant *in cis* duplication FSHD [30, 31], supporting the same diagnosis here.

In this study, four clinically FSHD-compatible, but genetically negative probands had an alternative diagnosis identified via exome analysis or single gene test; however, 25 probands remained genetically undiagnosed. The potential for false-positive genetic FSHD1 diagnoses is demonstrated by three 9U probands who, although technically FSHD1-diagnostic, each had a more appropriate alternative genetic diagnosis (Becker Muscular Dystrophy and FSHD2). For another 9U proband, FSHD severity seemed linked to a very rare homozygous 9U 4qA allele, as both heterozygous 9U parents were nonpenetrant. Caution is already exercised when interpreting 8-10U D4Z4 repeat arrays in European populations, also present in 1–2% of the healthy population: our data suggests great care must also be taken to rule out non-FSHD1 genetic causes in patients of Indian ancestry.

Our findings are timely, as more countries, including India, introduce OGM platforms with important potential to expand international testing for FSHD1 and other repeat disorders. Ongoing limitations may remain, however, in the form of prohibitively high costs of whole-family FSHD testing and limited access to FSHD2 methylation testing, as well as next-generation sequencing, muscle biopsy, and other tests to exclude similar conditions. It will be important for any site introducing FSHD genetic testing to acknowledge any relevant limitations and evaluate any genetic data in partnership with clinicians trained in applying standard FSHD scales and genetic data interpretation.

Overall, our study and those of others highlight several important unknowns [19–21]. We do not yet understand why populations from India, Japan and South Korea are less likely to manifest FSHD with 8-10U; there may be protective mechanisms in non-European populations and/or compounding factors in European populations. We also do not know if observed differences in susceptibility may impact FSHD treatment efficacy, underscoring the need for inclusive, global trials of emerging therapies. Comparable to other FSHD studies [33, 34], our clinically FSHD cohort had an FSHD genetic diagnostic rate of 67% (61/91). For the one-third to half of participants without a formal diagnosis after bespoke testing, further research is needed to clarify the range of conditions that phenocopy FSHD and to identify novel, pathogenic factors. We hope this cohort study and other international data will be harnessed to inform such research, and to deliver future, globally-relevant FSHD diagnostics and therapies.

### Supplementary information

Supplementary Material

## Data Availability

The data that support the findings of this study are available on request from the corresponding authors, in pseudonymised format. Depending on type of data requested and data processing planned, access may be subject to approval of the ICGNMD Data Access Committee and/or the AIIMS Ethics Committee.
