# Supplementary material for: The first genetically confirmed cohort of Facioscapulohumeral Muscular Dystrophy from Northern India
Source: Eur J Hum Genet. 2024 Apr 25;32(9):1053–64. doi: 10.1038/s41431-024-01577-z (PMC11368952; doi:10.1038/s41431-024-01577-z)
Supplement: Supplementary file 1 — Supplementary Material [file 41431_2024_1577_MOESM1_ESM.pdf]

Supplementary Figure 1: Flowchart summarizing study recruitment and genetic testing approach, with numbers tested.

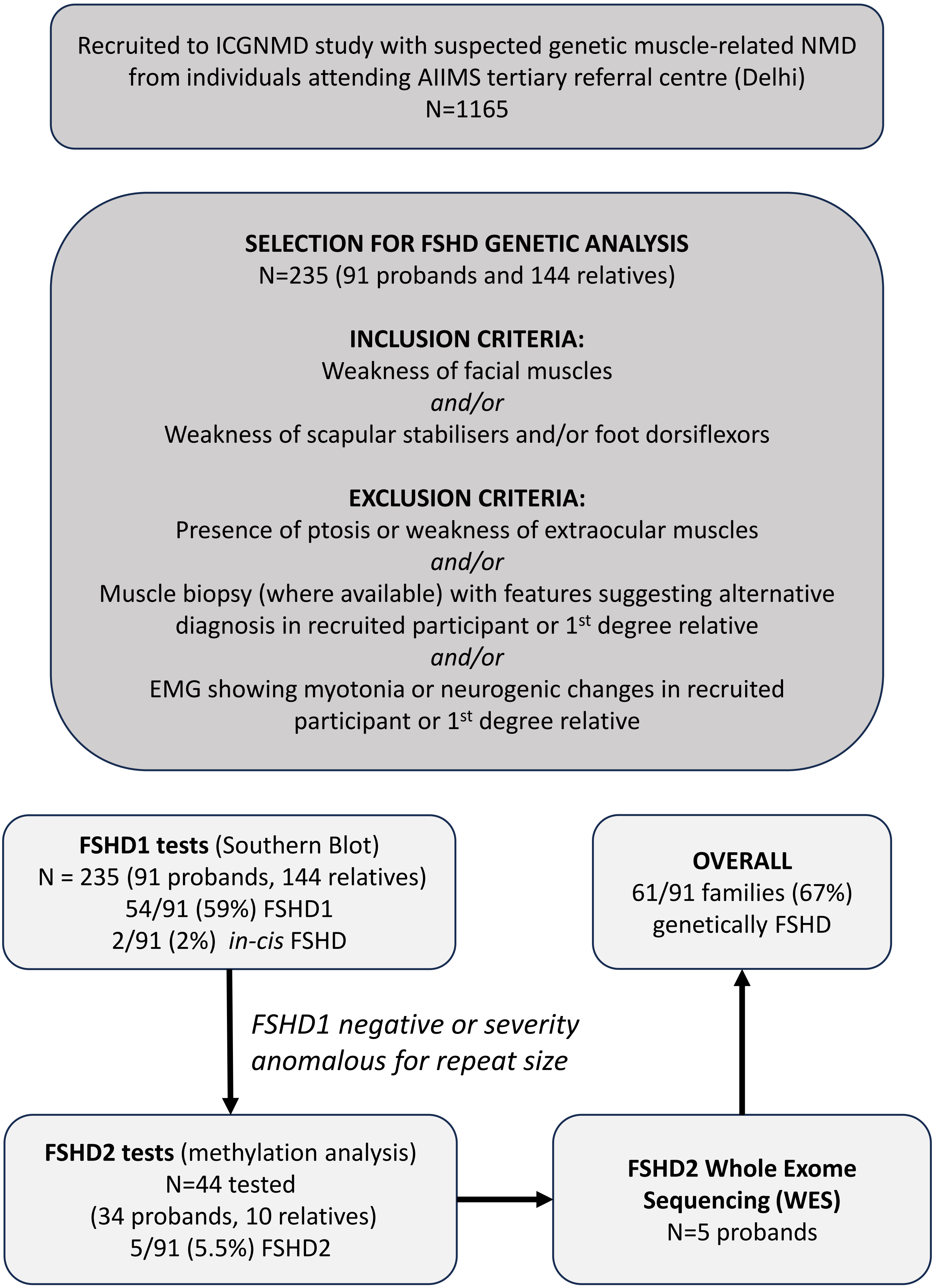

Supplementary Figure 2

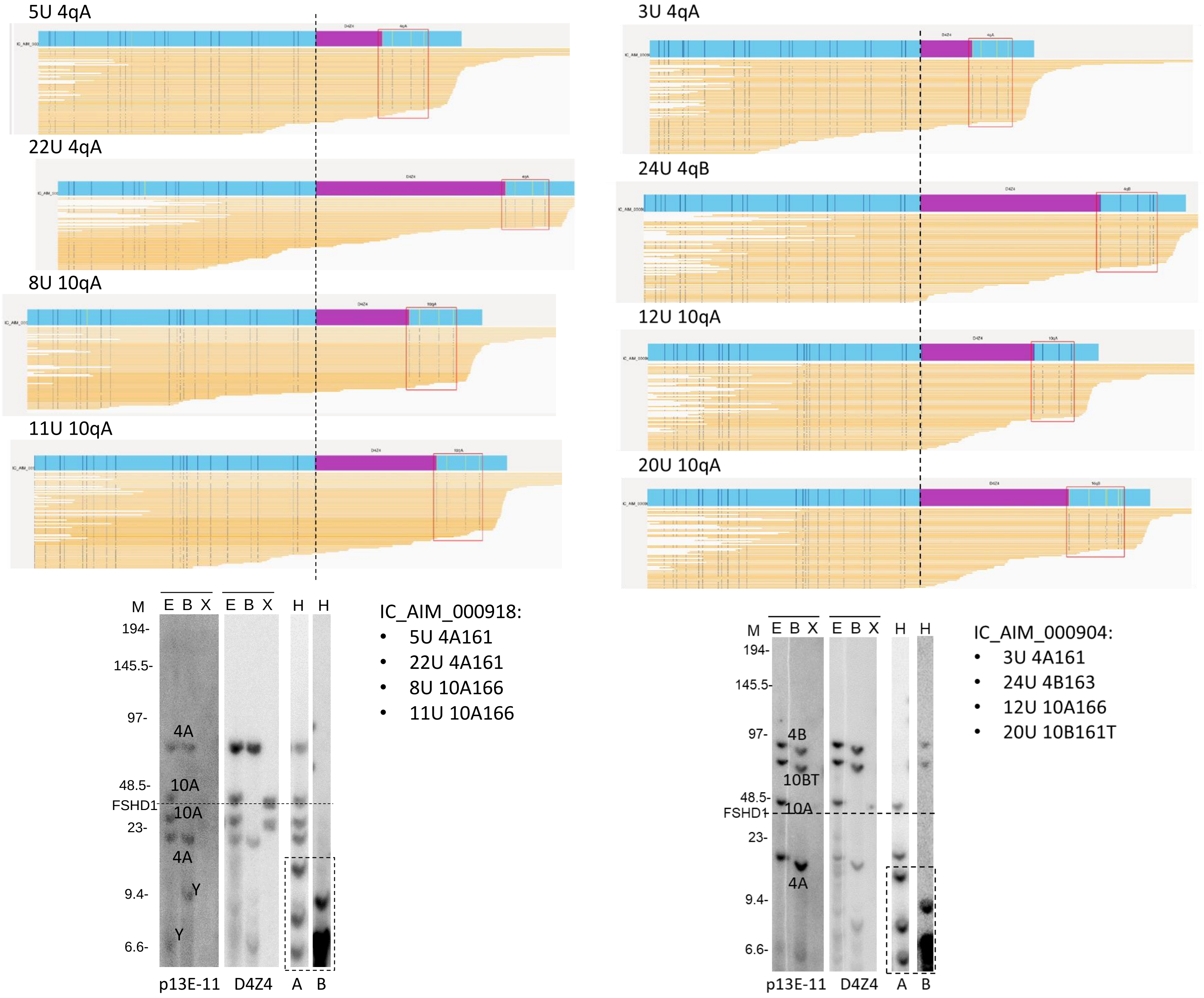

Previous page: **Supplementary Figure 2:** Comparison of optical genome mapping (top) and Southern blot hybridization and SSLP haplotyping (bottom) for the analysis of the probands IC_AIM_000918 and IC_AIM_000904. The optical genome mapping output files shows all reads aligned to the four D4Z4 alleles on chromosomes 4 and 10. Specific fluorescence tags proximal and distal to the D4Z4 arrays enable the chromosomal assignment and A-B haplotyping. The fluorescence tags are absent in the D4Z4 region and here the number of D4Z4 units is derived from the size of this unlabeled region. The Southern blot analysis shows hybridizations with probes p13E-11, D4Z4 on genomic DNA digested with enzymes EcoRI/HindIII (E), EcoRI/BlnI (B), XapI(X) and hybridizations with probes A and B on genomic DNA digested with HindIII (H). The alleles based on the different blots are indicated in the p13E-11 blot. The chromosome Y fragment in proband IC_AIM_000918 is indicated and the aspecific fragments in the A/B hybridizations are marked with a dotted box. The size of the molecular weight marker is indicated on the left and the threshold for FSHD (10U) is indicated by a dotted line.

Supplementary Figure 3

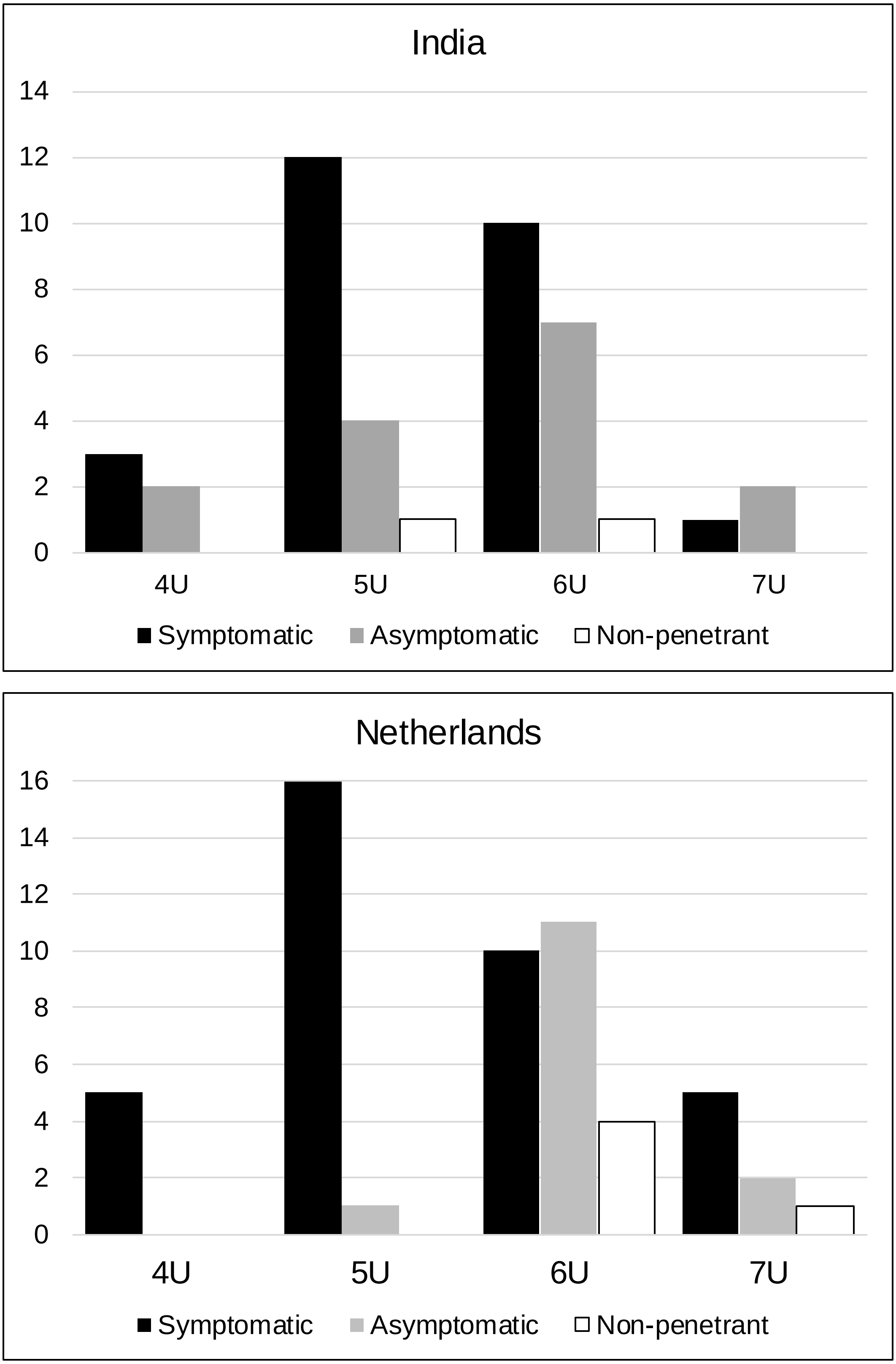

Supplementary Figure 3: D4Z4 repeat array size comparison of symptomatic carriers (black) and asymptomatic (grey) and non-penetrant carriers (white) of FSHD 4-7 Unit alleles among family members in our study cohort (9 families) compared to a previously-published Dutch cohort (10 families).[7] X axes: D4Z4 repeat array size, Y axes: number of individuals.

India						
	Gujariti Hapmap		our study		Total both cohorts	
**haplotype chr.4**	N	%	N	%	N	%
4A161L	1	0.6%	4	1.7%	5	1.3%
4A161S	66	42.9%	90	38.3%	156	40.1%
4A163		0.0%	5	2.1%	5	1.3%
4A166	2	1.3%	7	3.0%	9	2.3%
4A168		0.0%	1	0.4%	1	0.3%
4A166H	4	2.6%	9	3.8%	13	3.3%
4C161	1	0.6%	1	0.4%	2	0.5%
4B161	1	0.6%		0.0%	1	0.3%
4B162	1	0.6%	2	0.9%	3	0.8%
4B163	61	39.6%	96	40.9%	157	40.4%
4B166	2	1.3%	3	1.3%	5	1.3%
4B168	11	7.1%	12	5.1%	23	5.9%
4B170	4	2.6%	2	0.9%	6	1.5%
4B172			1	0.4%	1	0.3%
4B174			2	0.9%	2	0.5%
**total_4q**	154	100.0%	235	100.0%	389	100.0%
**haplotype chr.10**	N	%	N	%	N	%
DPED			1	0.3%	1	0.2%
10A164	1	0.6%	4	1.4%	5	1.1%
10A166	119	77.3%	225	77.6%	344	77.5%
10A166H	9	5.8%	13	4.5%	22	5.0%
10A168		0.0%	2	0.7%	2	0.5%
10A176T	1	0.6%	2	0.7%	3	0.7%
10A178T	1	0.6%	2	0.7%	3	0.7%
10B161T	22	14.3%	41	14.1%	63	14.2%
10C166	1	0.6%		0.0%	1	0.2%
**total_10q**	154	100.0%	290	100.0%	444	100.0%

Supplementary Table 1: Overview of the distribution of chromosome 4 and 10 haplotypes in worldwide populations with a different genetic background (Europe, Africa, East-Asia and India). The distributions in Europe, Africa and East Asia have previously been described and are added for comparison.5,8 The haplotype distribution in the healthy Indian population is based on non-FSHD related alleles in this study and on Gujarati Indians from the International HapMap project (https://www.genome.gov/10001688/international-hapmap-project). The frequency 4A161L in the total number of 4A161 alleles (S+L) is 8.2/(8.2+30.9) = 21.0% in Europe, and 1.3/(1.3+39.3) = 3.2% in India

**Europe**			
**haplotype chr.4**	**%**	**haplotype chr.10**	**%**
DPED	0.2	del10q	0.9
4A159	0.1	10A162	0.2
4A161L	8.2	10A163	0.1
4A161S	30.9	10A164	4.4
4A163	0.9	10A166	86.1
4A165	0.1	10A166H	0.6
4A166	4.4	10A176T	2.5
4A166H	3.9	10A180T	0.5
4A168	0.3	10B161T	4.6
4A170	0.2	Cohort: 96 from CEU 1000 Genomes 222 samples from Lemmers AJHG 2010 (data adapted from Lemmers et al AJHG 2010)	
4A172	0.2		
4B161	0.7		
4B162	1.8		
4B163	32.9		
4B166	1.0		
4B168	13.2		
4B170	0.5		
4B172	0.2		
4B174	0.2		

**East-Asia**			
**haplotype chr.4**	**%**	**haplotype chr.10**	**%**
DPED	2.8	10A164	25.8
4A161L	0.0	10A166	53.9
4A161S	34.3	10A166H	11.8
4A163	0.6	10B161T	8.4
4A166	0.6	Cohort: 96 from CHB/JPT 1000 genomes (data adapted from Lemmers et al AJHG 2010)	
4A166H	2.8		
4B163	57.3		
4B166	0.6		
4B170	0.6		
other	0.6		

**Africa**			
**haplotype chr.4**	**%**	**haplotype chr.10**	**%**
DPED	2.5	10A162	9.2
4A157	1.7	10A163	0.1
4A159	17.5	10A164	3.3
4A161L	0.0	10A166	77.5
4A161S	24.1	10A166H	3.3
4A165	0.1	10A176T	0.8
4A166	3.3	10A180T	1.7
4A166H	19.2	other	4.2
4B161	0.8	Cohort: 96 from YRI 1000 genomes (data adapted from Lemmers et al AJHG 2010)	
4B163	8.3		
4B166	0.8		
4B172	5.8		
4C166H	14.2		
other	1.7		

**India**			
**haplotype chr.4**	**%**	**haplotype chr.10**	**%**
4A161L	1.3	DPED	0.2
4A161S	40.1	10A164	1.1
4A163	1.3	10A166	77.5
4A166	2.3	10A166H	5.0
4A168	0.3	10A168	0.5
4A166H	3.3	10A176T	0.7
4C161	0.5	10A178T	0.7
4B161	0.3	10B161T	14.2
4B162	0.8	10C166	0.2
4B163	40.4	Cohort: 77 from Gujarati Indians 1000 genomes 110 samples from Indian cohort this study	
4B166	1.3		
4B168	5.9		
4B170	1.5		
4B172	0.3		
4B174	0.5		

			Clinical information					
**Individual**	**Relationship**	**Gender**	**AAE**	**Ricci (0-5)**	**ACSS (Ricci)**	**SUM score**	**ACSS (SUM)**	**Clinical Status**
IC_AIM_01050	father IC_AIM_01042	M	52	1.5	58	5	96	Asymptomatic
IC_AIM_01042	proband	M	19	4	421	11	579	Symptomatic
IC_AIM_01162	proband	M	18	2	222	6	333	Symptomatic
IC_AIM_01492	mother IC_AIM_01162	F	35	0	0	0	0	Nonpenetrant
IC_AIM_00817	proband	M	24	4.5	375	13	542	Symptomatic
IC_AIM_00932	proband	M	19	2	211	7	368	Symptomatic
IC_AIM_01192	proband	M	60	3	100	8	133	Symptomatic
	**shortest 4q**		**longest 4q**		**10_1**	**10_1**	**10_2**	**10_2**
**Individual**	**size (U)**	**haplotype**	**size (U)**	**haplotype**	**units101**	**SSLP101**	**units102**	**SSLP102**
IC_AIM_01050	19U	4A161S	21U	4B163	12U	10A166	36U	10A166duplication
IC_AIM_01042	19U	4A161S	19U	4A161S	36U	10A166dup	44U	10A166
IC_AIM_01162	15U	4A161S	17U	4A161S	16U	10A166	49U	10B161T
IC_AIM_01492	17U	4A161S	59U	4A161S	3U	10A166H1	49U	10B161T
IC_AIM_00817	9U	4A161S	34U	4B163	24U	10A166	29U	10A166
IC_AIM_00932	9U	4A161S	12U	4B163	38U	10A168	55U	10B161T
IC_AIM_01192	13U	4A161S	17U	4B174	11U	10A166	28U	10A166
	**SMCHD1 variant information**				**D4Z4 methylation**			
**Individual**	NM_015295.2	NP_056110.2	position	type	**FseI**	**delta1**		
IC_AIM_01050	not tested	not tested			4%	-38%		
IC_AIM_01042	c.823A>G	p.(Lys275Glu)	exon 7	Missense	6%	-41%		
IC_AIM_01162	c.1056C>G	p.(Tyr352Ter)	exon 9	Nonsense	8%	-34%		
IC_AIM_01492	not tested	not tested			12%	-34%		
IC_AIM_00817	c.1580C>T	p.(Thr527Met)	exon 12	Missense	15%	-29%		
IC_AIM_00932	c.3051_3075del	p.(Ser1017ArgfsTer20)	exon 25	Deletion	6%	-35%		
IC_AIM_01192	c.4101delA	p.(Lys1367fs)	exon 32	Deletion	11%	-27%		

Supplementary Table 2: Genetic characteristics of all genetically confirmed FSHD2 individuals (N=7) in our cohort. The individuals are from five unrelated families. Relationships within family and gender are indicated in columns 2 and 3 and clinical information in columns 4-9. D4Z4 repeat array size and haplotype is in columns 11-12. Columns 13-18 show the methylation levels found by FseI analysis and the calculated delta1 methylation (FSHD2 threshold is <-20%) and genetic information on the variant identified in SMCHD1 is in columns 20-25.

	Genetically Confirmed FSHD Negative Participants (N=30)		
	**Female**	**Male**	**Overall**
Total	6 (20%)	24 (80%)	30
Age at examination (yrs): median (range)	32.5 (24-47)	26 (10-57)	28 (10-57)
Consanguineous marriage	0	1/24= 4%)	1/30= 3%)
Age of onset of first symptom	26.5 (19-42)	21 (8-52)	22 (8-52)
Age of Diagnosis	32 (25-47)	25.5 (10-57)	27.5 (10-57)
Age of onset of diminished ability	24.5 (19-42)	20 (10-52)	20 (10-52)
Age of loss of ambulation	NA	1/24 (4%)	1/30 (3%)
**First ever symptom**	**Female**	**Male**	**Overall**
Facial muscle weakness	1/6 (17%)	0	1/30 (3%)
Proximal weakness in upper limbs	1/6 (17%)	4/24 (17%)	5/30 (17%)
Proximal weakness in lower limbs	3/6 (50%)	11/24 (46%)	14/30 (47%)
Proximal upper limb amyotrophy	0	2/24 (8%)	2/30 (7%)
Proximal lower limb amyotrophy	0	1/24 (4%)	1/30 (3%)
Scapular winging	0	2/24 (8%)	2/30 (7%)
Distal Lower limb muscle weakness	0	0	0
Distal UL muscle weakness	0	0	0
Foot Dorsiflexor weakness	0	0	0
Calf atrophy	0	1/24 (4%)	1/30 (3%)
Shoulder pain	1/6 (17%)	0	1/30 (3%)
**Clinical features**	**Female**	**Male**	**Overall**
Facial muscle weakness	5/6 (83%)	22/24 (92%)	27/30 (90%)
Proximal weakness in upper limbs	4/6 (67%)	16/24 (67%)	20/30 (67%)
Proximal weakness in lower limbs	3/6 (50%)	9/24 (38%)	12/30 (40%)
Scapular winging	4/6 (67%)	23/24 (96%)	27/30 (90%)
Distal weakness in lower limbs	2/6 (33%)	6/24 (25%)	8/30 (27%)
Distal weakness in upper limbs	1/6 (17%)	1/24 (45%)	2/30 (7%)
Lordosis/Scoliosis	1/6 (17%)	6/24 (25%)	7/30 (23%)
Beevor's sign	0	1/24 (4%)	1/30 (3%)
Hearing abnormality	0	1/24 (4%)	1/30 (3%)
Vision abnormality	0	0	0
Asymmetric Quadriceps weakness	0	0	0
Shoulder pain	1/6 (17%)	1/24 (4%)	2/30 (7%)
Diabetes Mellitus	0	1/24 (4%)	1/30 (3%)
Distal Hyperlaxity	0	0	0
Pectus excavatum	0	0	0
Muscle Biopsy	3/6 (50%)	4/24 (17%)	7/30 (23%)
Muscle MRI	1/6 (17%)	6/24 (25%)	7/30 (23%)
CK U/L	300(40-2358)	187 (58-17500)	229 (40-17500)
CK 500-1000	0	1/24 (4%)	1/30 (3%)
CK>1000	1/6 (17%)	6/24 (25%)	7/30 (23%)
Total MRC sum score (0-70), Median (range)	57 (49-69)	66(40-70)	65 (40-70)

Supplementary Table 3: Clinical and laboratory features of 30 FSHD negative cases. IC_AIM_00788 is included as, although a 9U FSHD1 allele is present, the diagnosis is considered Becker Muscular Dystrophy (BMD).
